# Virus-like Particle-Based L2 Vaccines against HPVs: Where Are We Today?

**DOI:** 10.3390/v12010018

**Published:** 2019-12-23

**Authors:** Rashi Yadav, Lukai Zhai, Ebenezer Tumban

**Affiliations:** 1 Department of Biological Sciences, Michigan Technological University, Houghton, MI 49931, USA; rashiy@mtu.edu (R.Y.); LZhai@mtu.edu (L.Z.); 2 Current address: Van Andel Research Institute, Grand Rapids, MI 49503, USA

**Keywords:** HPVs, vaccines, virus-like particles (VLPs), minor capsid protein (L2)

## Abstract

Human papillomaviruses (HPVs) are the most common sexually transmitted infections worldwide. Ninety percent of infected individuals clear the infection within two years; however, in the remaining 10% of infected individuals, the infection(s) persists and ultimately leads to cancers (anogenital cancers and head and neck cancers) and genital warts. Fortunately, three prophylactic vaccines have been approved to protect against HPV infections. The most recent HPV vaccine, Gardasil-9 (a nonavalent vaccine), protects against seven HPV types associated with ~90% of cervical cancer and against two HPV types associated with ~90% genital warts with little cross-protection against non-vaccine HPV types. The current vaccines are based on virus-like particles (VLPs) derived from the major capsid protein, L1. The L1 protein is not conserved among HPV types. The minor capsid protein, L2, on the other hand, is highly conserved among HPV types and has been an alternative target antigen, for over two decades, to develop a broadly protective HPV vaccine. The L2 protein, unlike the L1, cannot form VLPs and as such, it is less immunogenic. This review summarizes current studies aimed at developing HPV L2 vaccines by multivalently displaying L2 peptides on VLPs derived from bacteriophages and eukaryotic viruses. Recent data show that a monovalent HPV L1 VLP as well as bivalent MS2 VLPs displaying HPV L2 peptides (representing amino acids 17–36 and/or consensus amino acids 69–86) elicit robust broadly protective antibodies against diverse HPV types (6/11/16/18/26/31/33/34/35/39/43/44/45/51/52/53/56/58/59/66/68/73) associated with cancers and genital warts. Thus, VLP-based L2 vaccines look promising and may be favorable, in the near future, over current L1-based HPV vaccines and should be explored further.

## 1. Background

**Figure 1:**
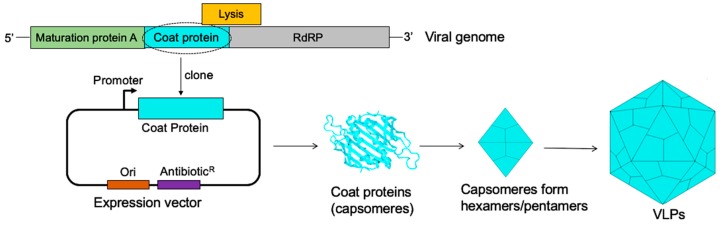
The assembly of bacteriophage MS2 coat proteins into VLPs (virus-like particles). The genome of MS2 bacteriophage (top image) with the coat protein (light blue). Cloning of the coat protein into a bacterial expression vector and expression of the protein in a suitable bacterial host gives rise to coat proteins (capsomers). The capsomeres form pentamers and hexamers; twelve pentamers and 20 hexamers spontaneously self-assemble to form a VLP (composed of 180 capsomeres). RdRP stands for RNA dependent RNA polymerase.

Virus-like particles (VLPs) are empty viral shells derived from the expression, in a suitable host cell, of viral structural proteins such as capsid or coat proteins. Over-expression of these proteins allows them to spontaneously self-assemble into VLPs (Figure 1). VLPs can also be derived from envelope proteins following over-expression of envelope proteins together with other structural proteins such as the pre-membrane proteins, capsid proteins, or over-expression of the envelope protein with Gag protein [1]. VLPs derived from the latter are known as enveloped VLPs. Thus, VLPs are empty shells which consist of one or more types of multimeric coat or envelope proteins arranged geometrically into dense, repetitive (multivalent) arrays [2,3,4]. They are morphologically and structurally similar to viruses from which the coat proteins are derived, except for the fact that they lack the viral genome. VLPs are highly immunogenic, even at small doses of antigens (as low as 500 ng) [5]. The following features have been credited for making VLPs highly immunogenic:

(i) Coat proteins that form the capsid are multivalently displayed to the immune system. Multivalent display enhances cross-linking of B-cell receptors on naïve B cells, leading to a stronger B-cell activation, proliferation, and differentiation, secretion of high-affinity antibodies, and the generation of long-lived memory B cells [6,7,8,9].
(ii) VLPs have virally encoded T-helper cell epitopes, which enhance T-cell activation. Presentation of these epitopes by antigen-presenting cells (APCs) in association with major histocompatibility complex class II to T-helper cells leads to the activation of T-helper cells. In addition to this, co-stimulatory molecules from APCs help to activate the T-helper cells. Activated T-helper cells then secret cytokines, leading to the activation of B-cells, T-cells, and macrophages [6].
(iii) Most VLPs are between 25 and 100 nm. This size range is very important for the following reasons. Firstly, nanoparticles between 10 and 200 nm, unlike those >200 nm, have been shown to efficiently enter the lymphatic system by direct diffusion through cell junctions. This allows VLPs to be efficiently exposed to immune cells. It is worth mentioning that VLPs can also be taken up and transported by APCs to the lymphatic system. Secondly, the small size of VLPs enables them to be transported to the lymphoid organs within a short period of time and to be efficiently taken up by APCs for presentation to T-helper cells [9].
(iv) Some VLPs (especially bacteriophage VLPs) contain single-stranded (ss)RNA, which serves as endogenous adjuvant. Bacteriophage coat proteins (from MS2, PP7, etc.) have the potential to encapsudate ssRNA that codes for its capsid/coat protein during VLP assembly [10,11]. This ssRNA serves as endogenous adjuvant by directly activating immunostimulatory molecules such as toll-like receptors (TLR7 and TLR8), which in turn activate innate immune responses [9,12,13,14].

The aforementioned features, in addition to the fact that VLPs are noninfectious and are safe, have made VLPs attractive biological agents for vaccine design and development. A number of VLP-based prophylactic vaccines have been approved by the Food and Drug Administration to protect against human papillomaviruses (HPVs) and hepatitis B virus (HBV) infections. Two VLP-based HBV vaccines are credited for reducing incidences of HBV-related hepatocellular carcinomas and mortalities, worldwide, with protection lasting for up to 30 years in some individuals [2,15]. On the other hand, three VLP-based HPV vaccines (Gadarsil-9, Cervarix, and Gardasil-4 (discontinued in the US)) have been approved within the last decade to protect against HPV infections.

## 2. HPV Vaccines

**Figure 2:**
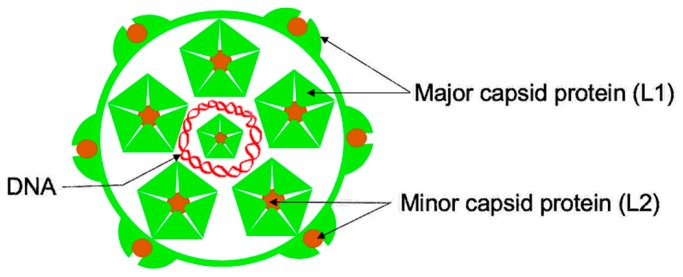
A schematic of HPV (human papillomavirus virion). A double-stranded DNA genome (red) is surrounded by the capsid, which is composed of two proteins: the major capsid protein (L1, shown in light green) and the minor capsid protein (L2, shown in brown color). The L1 protein forms pentamers, and L2 protein is inserted on vertices of the pentamers. Seventy-two copies of the pentamers and about 12–72 copies of L2 protein assemble to form a virion.

Approximately 42 HPV types can be transmitted sexually via anogenital to anogenital sex or anogenital to oral sex [16]. Out of these HPVs, ~19 types called high-risk types (oncogenic types; types 16, 18, 26, 31, 33, 35, 39, 45, 51–53, 56, 58, 59, 66, 68, 70, 73, and 82) are associated with cancers [17,18]. The remaining types, known as low-risk HPV types (types 6, 11, 40–44, 54, 61, 72, 81, etc.), are associated with genital warts and recurrent respiratory papillomatosis. VLP-based HPV vaccines have recently been shown to prevent cases of cervical intraepithelial neoplasias, with protection levels lasting for at least 10 years [19,20]. Moreover, recent studies show that a single dose of the HPV VLP-based vaccine can lead to long-lasting protection from HPV infection [21]. These vaccines are based on VLPs derived from over-expression of the capsid proteins in yeast (Gardasil vaccines) or in insect cells (Cervarix vaccine). HPV capsid is composed of two capsid proteins, the major capsid protein (L1) and the minor capsid protein (L2) (Figure 2). The L1 protein forms pentamers, and 72 copies of the pentamer assemble to form a capsid [22]. The L2 protein is suggested to be present as canyons at the vertices of pentamers and it is only transiently exposed following binding of the virion to heparan sulfate proteoglycans (HSPG) on the basement membrane [23,24]. The exact number of L2 protein on a virion is debatable. Studies suggest that about 12–72 copies of L2 proteins are present on a virion [25,26]. It is worth mentioning that L1 can assemble into the capsid without L2. L2 enhances encapsidation of a double-stranded circular DNA genome into the capsid, thus forming a virion [27]. The L2 protein also has other functions in the life cycle of the virus. Binding of a virion to HSPG promotes conformational change that exposes L2 on the capsid, and thus L2 enhances binding of the virion to epithelial cells [23,28]. It also promotes egress of the virion from the endosome [29] and trafficking of the viral genome towards the nucleus for replication [30,31].

**Figure 3:**
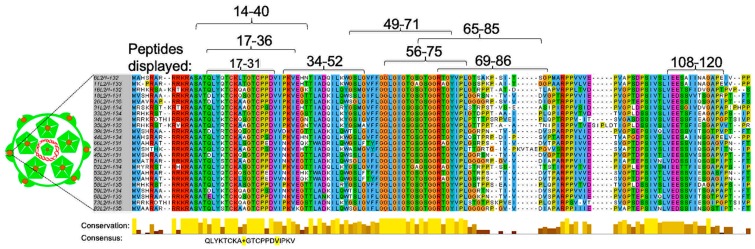
Sequence alignment of L2 (aa 1–135) from different HPV types. Sequence alignment was done using sequences from both high-risk (HPVs 16, 18, 26, 31, 33, 34, 35, 39, 45, 51–53, 56, 58, 59, 66, 68, 73, and 82) and low-risk HPV types (HPVs 6, 11, 43, and 44). Alignment was done using Jalview software. Residues that are highly conserved among different HPV types are highlighted, below the alignment, in gold bars. A consensus sequence of amino acid 17–36 from different HPV types is shown below the bars. Amino acid residues (in the consensus sequence) that differ from HPV16 L2 are highlighted in yellow background. The numbers above sequence alignment represent peptides (amino acid residues) that have been displayed on different VLP platforms (see Table 1 and Table 2 for details).

Although current L1-based HPV vaccines are highly immunogenic, they protect mostly against the HPV types included in the vaccines [32,33,34,35,36]; the L1 protein is not conserved among HPV types. For example, the most recent HPV vaccine (Gardasil-9, an upgrade of Gardasil-4) offers protection against seven HPV types (HPV16, 18, 31, 45, 33, 52, 58) associated with ~90% of cervical cancer and two HPV types (HPV6 and 11) associated with ~90% genital warts [17]. Thus, complete protection from HPV-associated cancers/warts may require the addition, to current HPV vaccines, of VLPs from HPV types that are not currently included in the vaccines. With this in consideration and given the fact that L1-based vaccines offer little cross-protection against other HPV types, the L2 protein has been explored within the last two decades to develop next-generation HPV vaccines. As shown in Figure 3, the L2 protein is conserved among different HPV types. This thus suggests that vaccines targeting L2 are going to be broadly protective against different HPV types. In fact, results from preclinical studies targeting L2 protein look very promising [37,38,39,40,41]. 

## 3. L2 Protein

Immunization with the N-terminus of L2 protein (out of the context of the L1 protein) elicits antibodies that protect and cross-protect against heterologous HPV types. For example, unlike antibodies elicited against L1 VLPs, antibodies elicited against an L2 peptide representing amino acids 1–88 from HPV type 18 or even from L2 of bovine papillomavirus (BPV) cross-protect, albeit at low titers, against diverse mucosal (HPV6, 11, 16, 18, 31) and cutaneous HPV types (HPV5) [37,38,39,40,41]. Given these data, efforts to develop next-generation HPV vaccines have focused on eliciting protective antibodies against L2 protein, especially against the first 130 amino acids of L2 peptide. Unfortunately, the L2 protein cannot form VLPs like L1 protein. Thus, studies to develop an HPV L2 vaccine have relied on using protein/peptide antigens. As mentioned earlier, L2 peptide antibody titers are very low. This could be explained by the fact that peptide antigens are very unstable in serum and are rapidly degraded following immunization [42,43]. As such, different strategies have been explored in preclinical studies to enhance protective antibody responses against L2 protein. For example, immunizations with an L2 peptide conjugated to thioredoxin [44,45], immunizations with concatemers of L2 protein fused to a self-adjuvanting protein (flagellin) [46,47] as well as immunizations with a concatemer of L2 proteins derived from different HPV types [48,49] have been used. More recently, an L2 polypeptide and a heptamerizing coiled-coil polypeptide OVX313 fused to a nanoparticle derived from a thermostable thioredoxin has been used [50,51,52]. These approaches have enhanced the immunogenicity of L2 peptides, especially immunization with the nanoparticle thioredoxin-L2-OVX313 candidate vaccine. The nanoparticle candidate vaccine offers protection against more than 14 HPV types [50]. Although the aforementioned strategies enhanced immune responses, the responses were observed mostly when large doses (up to 25 µg) of antigens, coupled with large amount of adjuvant with multiple immunizations regimens were used. With this in consideration, we and others have focused on immunizing with L2 peptides displayed multivalently on the surface of VLPs.

## 4. Multivalent Display of HPV L2 on VLPs

Not only can VLPs be used to develop vaccines against the virus from which the structural proteins are derived from, they can also be used as platforms to display heterologous antigens from other viruses [53], bacteria [54], pathophysiological diseases like cholesterol [55], and even tumor-associated antigens [56]. The goal of a chimeric VLP is to induce antibodies against a heterologous antigen displayed on the platform, but not against the platform. This technology has been exploited to develop VLP-based L2 vaccines against HPV, targeting some of the L2 peptides or epitopes shown in Figure 3. VLPs from viruses that infect bacteria (bacteriophages) or from viruses that infect eukaryotic cells have both been explored as platforms to display L2 peptides (Table 1 and Table 2).

### 4.1. The Display of L2 Peptides on Bacteriophage VLP Platforms

Different HPV L2 peptides (shown in Figure 3) have been displayed, by genetic insertion or by chemical conjugation, on VLPs derived from viruses (MS2, PP7, Qβ, AP205) that infect bacteria. Genetic insertion of L2 allows for DNA sequences that code for L2 peptides to be inserted by polymerase chain reaction on the single-chain dimer (two fused copies) of the coat proteins of bacteriophages MS2, PP7 or to be inserted on the coat protein monomer of bacteriophage AP205 in expression vectors (Table 1). Expression of the chimeric coat proteins in a suitable bacterial host allows 90 copies of the coat protein dimers (for MS2 and PP7) and 180 copies of coat protein monomers (AP205) to spontaneously assemble into VLPs, thus displaying 90 copies and 180 copies of L2 on the VLPs, respectively [5,11,57,58,59,60]. Chemical conjugation, on the other hand, allows chemically synthesized L2 peptides to be displayed mostly on Qβ VLPs as follows. Qβ VLPs are first expressed and purified from bacterial cells. Synthesized L2 peptides, carrying a terminal cysteine residue, are then cross-linked to lysine residues on the VLPs using a bi-functional cross-linker, succinimidyl 6-((beta-maleimidopropionamido)hexanoate) [61]. This allows for at least 360 copies of L2 to be displayed on Qβ VLPs.

As mentioned above, peptides representing different L2 epitopes have been displayed on bacteriophage VLPs. These peptides, which range in size from 12 amino acids (aa) to 110 aa, can be grouped into three categories based on the source of peptides:

(i) L2 peptides derived from different individual HPV types, especially HPV16 and HPV18. They correspond to aa 17–31, 20–29, 14–40, 34–52, 49–71, 56–75, 108–120, and so forth. [5,11,58,61].
(ii) L2 peptides derived from a concatemer of aa 17–31 and aa 20–31 from HPV16/HPV31 and a concatemer of aa 17–38 from five HPV types (HPV16, 18, 31, 35, 52) [57,60].
(iii) Peptides derived from a consensus sequence of aa 65–85 or aa 69–86 following the alignment of cancer-causing and wart-causing HPV types (Figure 3 and Table 1) [57,61].

Of all the above HPV L2 peptides that have been displayed on bacteriophage VLPs, only bacteriophage VLPs displaying peptides representing aa 17–31 from HPV5, 6, 16, 18 [5,58,59], bacteriophage VLPs displaying consensus aa 65–85 or 69–86 [57,61], and bacteriophage VLPs displaying peptides from the L2 concatemers (16L2/31L2 and 16L2/18L2/31L2/35L2/52L2) [57,60,62] elicited broadly neutralizing/protective antibodies against diverse HPV types (Table 1). For instance, immunization with a mixture of eight PP7 VLPs each displaying L2 peptide (aa 17–31) from eight different HPV types offered broader and better protection against HPV pseudoviruses 5/6/16/18/31/45/52/58 (Table 1) [58]. Each L2 peptide was inserted on the AB-loop of PP7 coat protein. Similarly, immunization with a mixture of two MS2–L2 VLPs (mixed MS2-L2 VLPs; one VLP displaying a concatemer peptide, aa 17–31 from 16L2 and aa 20–31 from 31L2 and another VLP displaying a consensus peptide from aa 69–86) offered robust broad protection against genital and oral infections with HPV pseudoviruses 11/16/18/31/33/35/39/45/52/53/56/58 (Table 1) [57,62]; the L2 peptides were inserted on the N-terminus of MS2 coat protein. Mixed MS2-L2 VLPs have the potential to protect against all eleven HPV types (tested so far) associated with ~95% of cervical cancer and against ~99% of HPV-associated head and neck cancers. Additionally, they can protect against one HPV type (HPV11, tested so far) associated with 36% of genital warts and ~32% of recurrent respiratory papillomatosis [57,62]. More HPV types need to be tested to assess the spectrum of protection. Thus, mixed MS2-L2 is an excellent next-generation candidate vaccine against HPV.

### 4.2. The Display of L2 Peptides on Eukaryotic VLP Platforms

L2 peptides have also been displayed on VLPs derived from eukaryotic viruses such as HPV, bovine papillomavirus type 1 (BPV1), adenovirus, adeno-associated virus, hepatitis B virus, and potato virus A (Table 2). All L2 peptides displayed on eukaryotic VLPs have been done by genetic insertion as described above. Inserted L2 peptides ranged from aa 17–36 (the most commonly inserted), aa 18–38, aa 56–75, aa 96–115, aa 108–120, aa 414–426, and so forth, and were derived mostly from HPV16. Inserted peptides were also derived from HPV31, HPV33, and HPV58 to a small extent (Table 2). The insertions have been made at different locations on the coat proteins. For example, on helix 4 loop (H4 helix, between aa 414/433 or between aa 430/433) on L1 coat protein of HPV16 (Table 2). Also, insertions have also been made between aa 136/177 (DE loop of L1 of HPV16), between aa 134/135 (DE loop of L1 of HPV18 and BPV1), and on the C-terminus of L1 coat protein of HPV18. Furthermore, insertions have also been made on some viruses/virus-associated particles. For example, insertions have been made on VP3 of Adeno-associated virus 2 virus particles at aa positions 587 and 453. Moreover, insertions have also been made on adenovirus types 5 and 35 (Table 2).

Of all these VLP insertions, HPV16 L1 VLPs displaying on the DE loop, L2 peptides representing aa 17–36 from HPV16 elicited antibodies that efficiently neutralized/protected against diverse HPV pseudovirus types (6/16/18/26/31/33/34/35/39/43/44/45/51/52/53/56/58/59/66/68/73) [69]. This is followed by HPV18 L1 VLPs displaying, on the DE loop and on the C-terminus, L2 peptides representing aa 17–36 from HPV33 and L2 peptides representing aa 56–75 from HPV58, respectively (Table 2). The VLPs protected rabbits from developing papillomas following challenge with diverse quasiviruses (6/11/16/18/31/35/39/45/59) [70]; the VLPs also protected mice from genital infection with HPV pseudoviruses (11/16/35/45/58/59). It is worth highlighting that HPV16 L1 VLPs displaying, between aa 130/433, L2 peptides representing aa 17–38 from HPV31 elicited antibodies that neutralized (to some degree) diverse HPV pseudovirus types (2/5/6/11/16/18/27/31/33/35/39/45/52/57/58/59/68) [72] (Table 2). Among the L2 insertions displayed on virus/virus-associated particles, Adeno-associated virus 2 VP3 virus particles displaying L2 peptides representing aa 17–36 from both HPV16 and HPV31 protected rabbits. The recombinant particles protected rabbits from developing papillomas following infection with eight different quasiviruses one year after immunization. Moreover, the rabbits did not develop papillomas for 10 weeks (the length of the study).

In summary, the type of peptide and the location of peptide insertion on eukaryotic VLPs make a difference in the neutralization potential. L2 peptide (aa 17–36) inserted on the DE loop elicits efficient broadly protective antibodies against diverse HPV types.

## 5. Expert Commentary and Perspectives for the Future

Current HPV vaccines based on L1 VLPs are highly immunogenic and offer robust protection against the HPV types included in the vaccines. However, they offer little cross-protection against non-vaccine HPV types [32,33,34,35,36]. Thus, complete protection against all HPV-associated cancers/warts requires the addition of VLPs from other HPV types not included in the vaccines, especially for Cervarix vaccine, which protects mostly against HPV16 and HPV18 (both HPV types are associated with ~70% of cervical cancer). From a production stand-point, the development and addition of more VLPs to current HPV vaccines may be costly. Also, although antigenic competition has not been reported for Gardasil-9 (with VLPs from nine HPV types), it is not clear whether the addition of more VLP types to the vaccine will lead to the immunodominance of certain VLP types. As an alternative to L1 vaccines, the L2 protein looks like an attractive target for a next-generation vaccine against HPV. Nevertheless, as mentioned above, L2 does not form VLPs and as such, it is less immunogenic. Fortunately, the display of peptides from L2 protein on VLPs enhances the immunogenicity of the peptides as well as its potential to protect against diverse HPV types. Although L2 peptides can be displayed by chemical conjugation or by genetic insertions, display by genetic insertion from a production standpoint seems to be the most cost-effective strategy. Constructs with genetically inserted L2 peptides can easily be expressed cheaply, at a large scale, in a host cell compared with chemical conjugation, whereby the peptides have to be synthesized commercially. Thus, chemical conjugation approach can be cost-prohibitive.

Among all the L2 peptides that have been displayed on VLPs, peptides representing amino acids 17–31 or 17–36 from HPV16 (and consensus sequence amino acid 69–86 to an extent) elicit antibodies with the broadest and the most efficient level of protection against diverse HPV types, regardless of whether a bacteriophage or eukaryotic VLP-platform was used to display the peptides. Within bacteriophage VLP platforms, MS2 VLPs displaying HPV16 L2 peptide (amino acids 17–31) elicit better cross-protection, while within eukaryotic VLP-platforms, HPV16 L1 VLPs displaying the HPV16 L2 peptide (amino acids 17–36) elicit better cross-protection. It is worth mentioning that the location of L2 insertion on the coat protein affects the immunogenicity of the peptide as well as the level of cross-protection. For bacteriophage VLPs, the insertion of amino acids 17–31 on the N-terminus seems to elicit better cross-protective antibodies against diverse HPV types compared to the same peptide inserted on the AB-loop ([5] and our unpublished data). While for HPV16L1 VLPs, the insertion of amino acids 17–36 on the DE loop seems to elicit better cross-protection against diverse HPV types [69] compared with insertions of same peptide on H4 helix (Table 2) [65,72].

**Figure 4:**
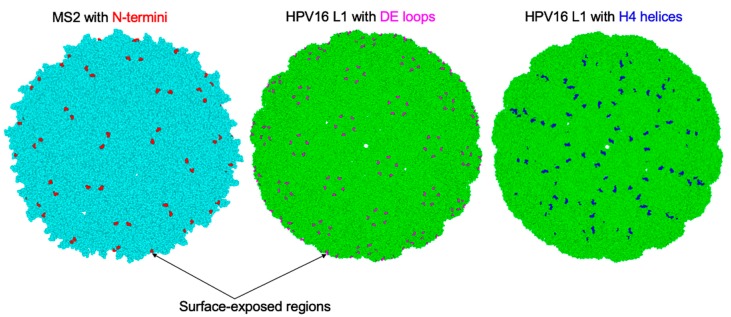
Model cryo-electron microscopy images of icosahedral structures of MS2 (light blue) and HPV16 L1 (light green) derived from protein data bank (PDB) with PDB identification numbers 2WBH and 5KEP, respectively. The N-termini on MS2 coat proteins where L2 peptides have been inserted are shown in red (left image). The DE loops on HPV16 L1 coat proteins where L2 peptides have been inserted are shown in magenta (middle image). The H4 helices on HPV16 L1 coat proteins where L2 peptides have been inserted are shown in blue (right image).

As already highlighted, peptides representing amino acids 17–31 or 17–36 from HPV16 (compared to the same peptide from other HPV types) elicit robust broadly protective antibodies against diverse HPV types (6/11/16/18/26/31/33/34/35/39/43/44/45/51/52/53/56/58/59/66/68/73) [57,62,67,68,69]. This is probably due to the fact that amino acid 17–36 from HPV16 is 90% identical to a consensus sequence of amino acid 17–36 (Figure 3). Thus, immunization with L2 peptide (amino acid 17–36) from HPV16 is almost like immunizing with a consensus peptide from this region. It is unclear why the insertion of this epitope on the N-terminus of MS2 VLPs as well as on the DE loop of HPV16L1 VLPs elicits robust cross-protective antibodies against diverse HPV types but marginal cross-protection when the epitope is inserted on H4 helix of HPV16L1 VLPs (Table 2). This could be explained by the location of the insertion and whether the whole peptide is readily displayed on the surface of the VLP and to the immune system. Modelled cryo-electron microscopy images of MS2 [79] and HPV16 L1 [80] seem to show that the N-termini of MS2 coat proteins and the DE loops of HPV16 L1 coat proteins (where the peptides are inserted) are readily exposed on the surface of their icosahedral structures compared with the H4 helix of HPV16 L1 (Figure 4). In addition to this, insertions at these different locations can affect the assembly of the chimeric coat proteins into VLPs and ultimately, their immunogenicity. Transmission electron microscopy images of chimeric MS2 VLPs and HPV16 L1 VLPs with L2 peptide amino acid 17–36 insertions at the N-terminus and DE loop, respectively, show VLPs with conformations similar to their respective wild-type VLPs [5,67]. However, insertion of the same peptide on the H4 helix of HPV16 L1 coat protein gives rise to VLPs, some of which have irregular conformations and difference sizes [65]. Thus, the location of the insertion and/or the display of L2 peptide on the surface of a VLP platform is important in eliciting robust broadly protective antibodies against diverse HPV types. In summary, the N-terminus of MS2 and the DE loop of HPV16 L1 seem to be optimal locations to display L2 peptides, especially peptide 17–36.

Overall, VLP-based L2 vaccines against diverse HPV infections look promising and may be favorable, in the near future, over current L1-based HPV vaccines. HPV16 L1-16L2 (aa 17–31) VLPs and mixed MS2-L2 VLPs ((MS2–16L2/31L2 and MS2–consL2 (69–86)) can be formulated as a monovalent vaccine [67,69] or bivalent vaccine [57,62], respectively, compared with current HPV vaccines (especially the nonavalent Gardasil-9). HPV16 L1 VLPs displaying peptide 17–36 on the DE loop neutralize/protect against sexually transmitted HPVs associated with cancers/warts as well as against non-sexually transmitted HPV types (HPV 3, HPV5) associated with cutaneous warts [67,69]. On the other hand, mixed MS2-L2 VLPs have been shown to protect, so far, against sexually transmitted HPVs associated with cancers/warts [57,62]. Mixed MS2-L2 VLPs have also been assessed against oral HPV infection, and the results look promising.

Although aforementioned preclinical protective studies look promising, there are still some questions that remain to be answered with both candidate vaccines. For example, we still do not know if immune responses elicited by HPV16 L1-16L2 (aa 17–31) VLPs or mixed MS2-L2 VLPs will last 3–8 years, as has been shown with a single dose of Gardasil-4 [21]. Recent preclinical studies with four doses (25–50 μg/dose) of HPV16 L1-16L2 (aa 17–31) VLPs show that the antibodies are protective 1 year after immunization [69]. Ten micrograms/dose (two doses total) of MS2 VLPs displaying peptide 17–36 has been shown in preclinical studies to be protective 18 months after immunization [81]. It is yet to be seen how long immune responses elicited by the mixed MS2-L2 will last. Thus, studies are required to assess the longevity of protection at different doses for these VLP-based L2 candidate vaccines.

Additionally, studies are required to assess the thermostability of candidate vaccines. Gardasil-9 has recently been shown to be thermostable at temperatures of up to 40 °C for three months [14,82]. Insertion of peptides on VLPs has been shown to decrease the thermostability of chimeric VLPs [83]. Thus, it would be nice to know if these candidate VLP-based L2 vaccines are thermostable given the fact that refrigeration and temperature-monitoring facilities are underdeveloped in developing countries, which have the highest burden of HPV infection and are in dire need of the vaccines. If the candidate vaccines are not thermostable, their thermostability can be enhanced by spray drying or spray freeze-drying. We have recently shown that spray freeze-dried mixed MS2-L2 VLPs stored at room temperature for two months are thermostable and protective [62].

Studies are also required to assess the effect of pre-existing L1 antibodies (from natural infection) to the immunogenicity of HPV16 L1-16L2(aa 17–31) VLPs. Studies have shown that pre-existing antibodies to some platforms can reduce the immunogenicity of the platforms and consequently, the immunogenicity of peptide displayed on the platforms [84,85,86,87,88,89]. This may not be an issue with mixed MS2-L2 VLPs given the fact that antibodies against MS2 (including PP7 or Qβ bacteriophages) have never been detected in humans. We have not come across any data documenting the presence of antibodies against these viruses in humans. Taken together, VLPs derived from bacteriophages (MS2) or eukaryotic viruses (HPV16 L1) displaying L2 peptides have the potential to serve as next-generation vaccines against HPVs and should be explored further.

## Figures and Tables

**Table 1:** Candidate bacteriophage VLP-based L2 HPV vaccines.

Source of VLPs	HPV L2 Epitope Displayed (aa)	Length of Epitope	Position of Insertion on VLPs	Dose Immunized with	Neutralized or Protected against HPV Pseudoviruses (PsVs) *	Ref ^#^.
MS2	HPV16 L2 (aa 17–31)	15	N-terminus	Mice immunized with two doses of VLPs (5 μg/dose)	PsVs (genital): 5/**6**/**16**/33/**35**/39/**45**/51/53/58	[5]
	HPV16 L2 (aa 20–31)	12	‘’	‘’	Not tested	
	HPV16 L2 (aa 14–40)	26	‘’	‘’	‘’	
	HPV16 L2/31L2 (aa 20–31 + aa 17-31)	27	N-terminus	Mice immunized with two doses of a mixture of VLPs displaying 16L2/31L2 and VLPs displaying consensus epitope (10 μg/dose)	PsVs (genital): **16**/**18**/**31**/**33**/**45**/58	[57]
	Consensus (aa 69–86)	18		Mice orally immunized with three doses of a mixture of VLPs displaying 16/31L2 and VLPs displaying consensus epitope (100 μg/dose)	PsVs (genital): 11/**16**/**53**/**56**	[62]
					PsVs (oral): **16**/**35**/**39**/**52**/58	
	HPV16 L2 (aa 17–31)	15	16L2 on N-terminus of one coat protein and 31L2 on the N-terminus of another coat protein	Mice immunized with two doses of hybrid VLPs (5 μg/dose)	PsVs (neutralized): **16**/**31**/**45**/**18**/**58**	[59]
	HPV31 L2 (aa 17–31)	15				
PP7	HPVs: 1L2, 5L2, 6L2, 11L2, 16L2, 18L2, 45L2, 58L2 (aa 17–31)	15 from each L2 on each VLP	AB-loop (internal insertion)	Mice immunized with three doses of a mixture of VLPs displaying all eight L2 peptides (10 μg/dose)	PsVs (genital): 6/16/**18**/**31**/**45**/**52**/58 PsVs (cutaneous): **5**	[58]
PP7	HPV16 L2 (aa 17–31)	15	16L2 on AB-loop of one coat protein and 18L2 on the AB-loop of another coat protein	Mice immunized with two doses of hybrid VLPs (5 μg/dose)	PsVs (genital): 6	[59]
	HPV18 L2 (aa 17–31)	15				
	HPV16 L2 (aa 35–50)	16	Chemical conjugation on surface	Mice immunized with three doses each VLP (5 μg/dose)	PsVs (genital): 16 (no protection)	[61]
	HPV16 L2 (aa 51–65)	15			PsVs (genital): 16	
Qβ	HPV16 L2 (aa 34–52)	19	Chemical conjugation on surface	Mice immunized with three doses of each VLP (5 μg/dose)	PsVs (genital): 16 (no protection)	[61]
	HPV16 L2 (aa 49–71)	23			PsVs (genital): 16	
	Consensus (aa 65–85)	18			PsVs (genital): **16**	
	HPV16 L2 (aa 108–120)	13			PsVs (genital): 6/**16**/31/45/**58**	
AP205	A concatemer of HPV: 16L2, 18L2, 31L2, 35L2, 52L2 (aa 17–38)	110	C-terminus	Mice immunized with two doses of VLPs (5 μg/dose)	PsVs (genital): 16, 31, 35, 45, **52**	[60]

* Bolded PsVs indicate that protection/neutralization was both significant and complete, while PsVs not bolded (in black) indicate that although the protection/neutralization was significant, it was not complete. PsVs not bolded (in gray background) indicate that there was minimal protection/neutralization or no protection at all (not significant protection); ^#^ Ref: Reference.

**Table 2:** Candidate eukaryotic VLP-based L2 HPV vaccines.

Source of VLPs	HPV L2 Peptide Displayed (aa)	Length of Peptide	Position of Insertion on VLPs	Dose Immunized with	Neutralized or Protected against HPV Pseudoviruses (PsVs) *	Ref ^#^.
HPV16 L1	HPV16 L2 (aa 414–426)	13	Helix 4 loop (aa 414–426 replaced with L2)	Mice immunized with three doses of VLPs (100 μg/dose)	Assays not conducted; however, the peptide has been shown, in previous studies [63], to neutralize HPV 16 and HPV 6	[64]
HPV16 L1	HPV16 L2 (aa 18–38)	21	Helix 4 loop (between amino acids 430 and 433)	Rabbits immunized with five doses of VLPs (concentration not provided)	PsVs (neutralized): **16**/18/**31**/52/58	[65]
	HPV16 L2 (aa 56–75)	20			PsVs (neutralized): **16**/18/31/**52**/58	
	HPV16 L2 (aa 96–115)	20			PsVs (neutralized): **16**/18/31/52/58	
BPV1	HPV16 L2 (aa 69–81)	13	Between amino acids 133 and 134 (DE loop)	Rabbits immunized with three doses of VLPs (50 μg/dose)	PsVs (neutralized): PsV11/PsV16	[66]
	HPV16 L2 (aa 108–120)	13			PsVs (neutralized): PsV11/PsV16	
BPV1	HPV16 L2 (aa 17–36)	20	Between amino acids 133 and 134	Rabbits immunized with four doses of VLPs (25–50 μg/dose)	PsVs (neutralized): **5**/**16**/18/45/58	[67]
HPV16 L1	HPV16 L2 (aa 17–36)	20	Between amino acids 136 and 137 (DE loop)	Rabbits immunized with four doses of VLPs (25–50 μg/dose)	PsVs (neutralized): 5/6/11/**16**/18/**31**/45/52/58/70	[67,68]
					Passive transferred sera protected mice against PsVs (genital): **6**/**16**/**18**/**26**/**31**/**33**/**34**/**35**/**39**/**43**/**44**/**45**/**51**/**52**/**53**/**56**/**58**/**59**/**66**/**68**/**73**	[69]
				Mice immunized with two doses of a mixture of 16L1 VLPs displaying 16L2 and HPV18 L1 VLPs (10 μg/dose)	PsV (genital): **58**	[68]
HPV18 L1	HPV33 L2 (aa 17–36)	20	Between amino acids 134 and 135 (DE loop)	Rabbits immunized with three doses of each VLP (20 μg/dose or 100 μg/dose)	QsV ^$^ (neutralized): 5/6/**11**/**18**/16/31/33/45/52/**58** QsVs (cutaneous): **11**/**18**/**58**	[70]
	HPV58 L2 (aa 56–75)	20	C-terminus		QsV (neutralized): 31/45	
HPV18 L1	HPV33 L2 (aa 17–36)	20	HPV33 L2 inserted on DE loop and HPV58 L2 inserted on C-terminus	Rabbits immunized with three doses of a mixture of 18L1 VLPs displaying 33L2 on DE loop and 58L2 on C- terminus mixed human dose of 16L1/18L1 VLPs (Cervarix vaccine). Two μg/dose of chimeric 18L1-L2 and 1/10th per dose of Cervarix was used	QsVs (cutaneous): 6/**11**/**16**/18/**31**/**35**/**39**/**45**/59	[70]
	HPV58 L2 (aa 56–75)	20		Mice immunized with two of a mixture of 18L1 VLPs displaying 33L2 on DE loop and 58L2 on C- terminus mixed human dose of 16L1/18L1 VLPs (Cervarix vaccine). Two μg/dose of chimeric 18L1-L2 and 1/10th per dose of Cervarix was used	PsVs (genital): **11**/**16**/**35**/**45**/**58**/**59**	
HPV18 L1	HPV45 L2 (aa 16–35)	20	Between amino acids 134 and 135	Rabbits immunized with five doses of VLPs (50 μg/dose)	Passive transferred sera protected mice against PsVs (genital): 16/**18**/39/**45**/59/**68**	[71]
HPV16 L1	HPV31 L2 (aa 17–38)	22	Helix 4 loop (between amino acids 430 and 433)	Mice immunized with four doses of VLPs (10 μg/dose)	PsVs (neutralized): 2/5/6/11/**16**/18/27/**31**/33/35/39/45/52/57/58/**59**/68	[72]
Adeno- associated virus 2 VP3 virus particles	HPV16 L2 (aa 17–36)	20	HPV16 L2 inserted at position 587 and HPV31 L2 inserted at position 453 of VP3	Mice immunized with three doses of particles (1 × 10^11^ per dose)	PsVs (neutralized): **16**/18/**31**/45/52/58	[73]
	HPV31 L2 (aa 17–36)	20		Rabbits immunized with four doses of particles (2 × 10^12^ per dose)	PsVs (neutralized): **16**/**18**/**31**/45/52/58 Passive transferred sera protected mice against PsVs (genital): **16**	
Adeno- associated virus 2 VP3 virus particles	HPV16 L2 (aa 17–36)	20	HPV16 L2 inserted at position 587 and HPV31 L2 inserted at position 453 of VP3	Mice immunized with three doses of particles (1 × 10^12^ per dose)	PsVs (neutralized): **16**/**18**/31/45/58	[74]
	HPV31 L2 (aa 17–36)	20		Rabbits immunized with three doses of particles (1 × 10^19^ per dose)	QsVs (cutaneous): **16**/**18**/**31**/**35**/**39**/**45**/**58**/**59**	
Adenovirus type 5	HPV16 L2 (12–41)	30	Hexon protein hypervariable regions 1 and 5	Mice immunized with three doses of particles (1 × 10^10^ per dose)	PsVs (genital): **16**/56 PsVs (neutralized): 16/73	[75]
Adenovirus type 35	Concatemers of HPV: 6L2, 11L2, 16L2, 18L2, 31L2, 33L2, 45L2, 52L2, 58L2 (aa 17–36)	60, 80, 100	C-terminus of pIX	Mice immunized with two doses of particles (1 × 10^10^ per dose)	PsVs (neutralized): **16**/**18**/31/59	[76]
Hepatitis B core (HBc) VLPs	HPV16 L2 (aa 14–122)	108	Inserted between a heterodimer of HBc	Mice immunized with three doses of VLPs (5μg/dose)	PsVs (neutralized): **16**	[77]
Potato virus A VLPs	HPV16 L2 (aa 108–120)	13	L2 inserted on the N-terminus and E7 inserted on the C-terminus	Immunization studies not conducted	No studies	[78]
	HPV16 E7 (aa 44–60)	17				

* Bolded PsVs indicate that protection/neutralization was both significant and complete, while PsVs not bolded (in black) indicate that although the protection/neutralization was significant, it was not complete. PsVs not bolded (in gray background) indicate that there was minimal protection/neutralization or no protection at all (not significant protection); ^#^ Ref: Reference; ^$^ QsV: quasivirus (composed of HPV capsid and cottontail rabbit papillomavirus genome).
